# Bioactive Compounds Isolated from Neglected Predatory Marine Gastropods

**DOI:** 10.3390/md16040118

**Published:** 2018-04-05

**Authors:** Ashlin H. Turner, David J. Craik, Quentin Kaas, Christina I. Schroeder

**Affiliations:** Institute for Molecular Bioscience, The University of Queensland, Brisbane, 4072, Qld, Australia; ashlin.turner@imb.uq.edu.au (A.H.T.); d.craik@imb.uq.edu.au (D.J.C.)

**Keywords:** toxins, marine gastropods, salivary glands, peptides, Ranellidae, echotoxins

## Abstract

A diverse range of predatory marine gastropods produce toxins, yet most of these molecules remain uncharacterized. *Conus* species have received the most attention from researchers, leading to several conopeptides reaching clinical trials. This review aims to summarize what is known about bioactive compounds isolated from species of neglected marine gastropods, especially in the Turridae, Terebridae, Babyloniidae, Muricidae, Buccinidae, Colubrariidae, Nassariidae, Cassidae, and Ranellidae families. Multiple species have been reported to contain bioactive compounds with potential toxic activity, but most of these compounds have not been characterized or even clearly identified. The bioactive properties and potential applications of echotoxins and related porins from the Ranellidae family are discussed in more detail. Finally, the review concludes with a call for research on understudied species.

## 1. Introduction

Predatory marine gastropods are at a distinct disadvantage when attempting to catch larger, faster, or more agile prey; but they have evolved potent venoms to subdue their prey to compensate for their small size and slow pace [1,2]. The most studied marine gastropod venoms are those of the Conidae, also called *Conus* species and commonly named cone snails, which use a harpoon-like radula tooth to deliver potent neurotoxins. Different *Conus* species feed on different prey, ranging from small fish to worms and even other gastropods [3,4,5]. The venom of each of the >700 cone snail species comprises a complex mixture of hundreds to thousands of peptides, called conopeptides; most of them target specific ion channels and transporter subtypes of the nervous system [1,2,6]. Cone snail venoms display large variability between species and even between individuals of the same species, and the total pool of cone snail peptides is estimated to be in the hundreds of thousands [7,8,9]. For more than 40 years, cone snail venoms have been mined for drug-lead compounds, the most well-known being conopeptide MVIIA (ziconotide), which is used clinically for treating intractable pain [10]. Several other conopeptides have entered clinical trials with varying levels of success [1].

Whereas *Conus* species have been in the spotlight of drug discovery, several other marine gastropod species are also potential sources of pharmaceutically attractive bioactive compounds. For example, while true venom glands are limited to (but not ubiquitous in) the superfamily Conoidea [11], some species from the Tonnoidea superfamily have indeed demonstrated the ability of their accessory salivary glands to function as ‘venom’ glands [3,12]. Most species of predatory gastropods in Tonnoidea have a large pair of salivary glands that are differentiated into anterior and posterior lobes, and are responsible for producing sulfuric acid (pH 2) [13,14]. 

**Figure 1:**
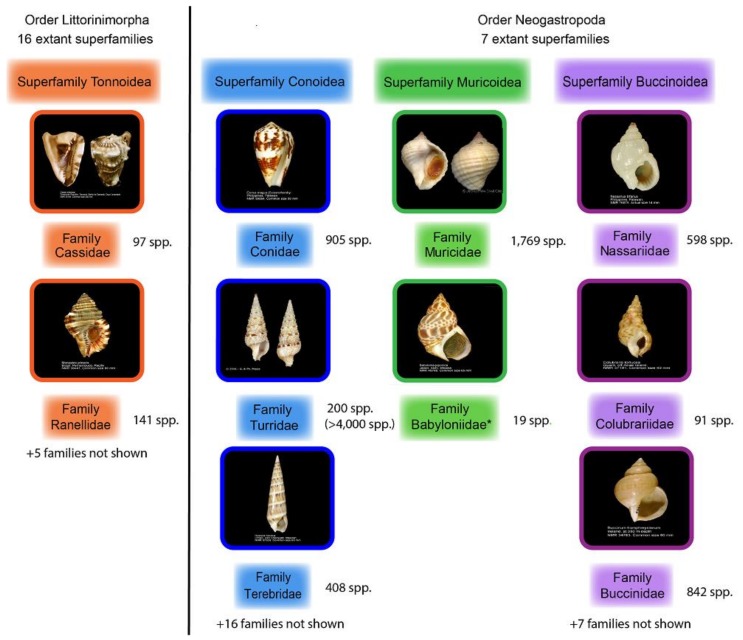
Families from Subclass Caenogastropoda that are covered in this review. Family and species numbers are current as listed from the WoRMs database and represent confirmed species within that family, whereas the species numbers in brackets represent an estimate of the total diversity from published literature [16]. Photographs are from the WoRMs database and provided under the Creative Commons License [17]. * The classification of the family Babyloniidae is currently under review by the ICZN [17].

This review aims to summarize what is known from neglected families of carnivorous gastropods (Figure 1, Table 1); starting with a broader view of known poisonous marine gastropods, and then focusing on the understudied Ranellidae family. It provides an update to the Modica and Holford review published in 2010 [15], similarly focusing on bioactive substances found in Neogastropoda, but also expanding the scope to include other interesting but understudied gastropod families. All cited taxonomy follows the World Register of Marine Species (WoRMs). 

## 2. Bioactive Compounds Isolated from Diverse Predatory Gastropods 

### 2.1. Terebrids—Auger Snails

Besides *Conus*, a subset of the closely related family Terebridae (terebrids) also possesses a specialized radula, used as a spear or harpoon to deliver potent neurotoxins. These ~300 known species fall into three feeding types [18]: type I species have salivary glands, an eversible labile tube, short buccal tube, and lack venom apparatus; type II species, the most similar to *Conus*, have true venom glands and a delivery apparatus in the form of a specialized radula; and type III lack a venom apparatus, but have an accessory proboscis that other terebrids lack [11,19]. Several type II species have a venom that exhibits similarities to conopeptides. These members of Terebridae produce small peptides with multiple disulfide linkages, named “teretoxins” [20,21]. Although bearing superficial similarities to conopeptides, there are several differences between teretoxins and conopeptides; most notably they have distinct sequences, degree of post-translational modifications and some of their cysteine frameworks were not found in conopeptides [18]. 

**Figure 2:**
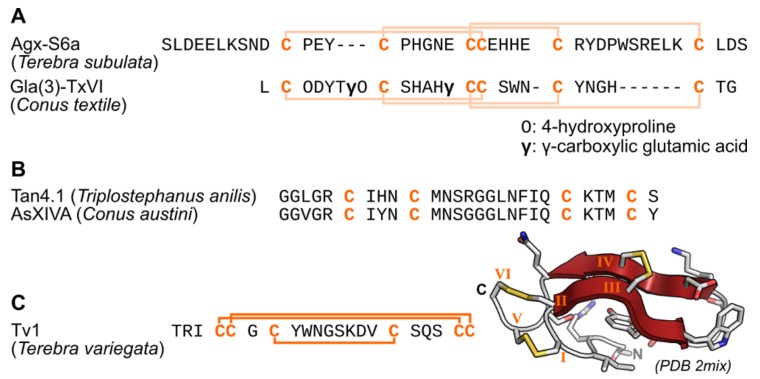
Sequence and structure of selected teretoxins. (**A**) Comparison of the mature toxins of teretoxin Agx-S6a to the conopeptide Gla(3)-TxVI [24], which is the closest conopeptide to Agx-S6a in the ConoServer database [23]. (**B**) Comparison of the predicted mature sequence of teretoxin Tan4.1 to the sequence of conopeptide AsXIVA [25], which share 85% similarity. (**C**) Sequence and structure of teretoxin Tv1 solved by nuclear magnetic resonance solution [26]. The species from which each peptide originates is indicated in parentheses. The Cys residues are in orange. Disulfide connectivities predicted by homology are indicated by light orange lines and experimentally verified connectivities are represented as dark orange lines. The N and C termini are indicated on the structure of Tv1 and the β-sheet is colored in red. The Cys residues are numbered using Roman numerals on the three-dimensional structure.

From *Terebra subulata*, three teretoxins (Agx-S6a, Agx-S7a, AgxS11a) were purified and demonstrated no posttranslational modifications besides the formation of disulfide bonds, whereas conopeptides are typically heavily posttranslationally modified [22]. These toxins were highly divergent from conopeptides but display a similar cysteine framework, as illustrated in Figure 2. In a later study, eight teretoxins were purified from *Hastula hectica*, and these toxins also displayed no posttranslational modifications [18]. Interestingly, several enzymes involved in posttranslational modifications of peptides were discovered in the transcriptome of Terebra venom glands, suggesting that teretoxins can display a range of posttranslational modifications. Identified enzymes in *Cinguloterebra anilis* (previously known as *Triplostephanus anilis*) and *Terebra subulata* include γ-glutamyl carboxylase, peptidyl-glycine α-amidating monooxygenase, prolyl-4-hydroxylase, tyrosyl-sulfotransferase, and glutaminyl-peptide cyclotransferase [21]. Especially, the γ-glutamyl carboxylase enzyme catalyzes the γ-carboxylation of Glu into Gla, which is a frequent modification of conopeptides [23]. Such modification could not be detected in the venom of *Hastula hectica* using Gla-specific monoclonal antibodies, supporting that teretoxins are generally less post-translationally modified than conopeptides [18]. 

The largest study of terebrid venom came from a transcriptomics study of *Cinguloterebra anilis* and *Terebra subulata* in which 139 putative teretoxins were identified [21]. These toxins display highly variable sequences and a high diversity of cysteine frameworks, suggesting a range of neural targets. The organization of the teretoxin transcripts was remarkably similar to that of cone snail toxins, with a signal peptide and a mature peptide region, which is excised from a precursor region. Conopeptides are classified into gene superfamilies according to the sequence similarity of their signal peptides, and a similar classification was attempted for this set of terebrid transcripts, leading to the identification of 14 gene superfamilies [21]. The consensus signal peptide sequences defining these superfamilies were generally different from those of the conopeptide gene superfamilies with the exception of the TM terebrid gene superfamily, which displayed 80% sequence identity in its signal sequence to the H conopeptide gene superfamily. In this study, twelve cysteine frameworks were discovered, ten of which were already described for conopeptides, but two were novel cysteine frameworks [21]. All teretoxins predicted from these transcriptomic sequences had no sequence similarity with known conopeptides with the exception of one teretoxin, Tan14.1, which has marked similarity to conopeptide AsXIVa (Figure 2). 

Cysteine frameworks are strongly associated with the three-dimensional conformation of disulfide-rich peptides and their function. Nevertheless, the cysteine framework classification does not consider the connectivity between Cys, which is difficult to determine, but only their pattern in the toxin primary sequence, and the cross-links created by disulfide bonds are a major determinant of the fold of small peptides. Therefore, teretoxins displaying novel cysteine frameworks [21] have a high probability of adopting novel folds, but even toxins displaying a framework identified in cone snail toxins could display a different disulfide connectivity and therefore different structure. This is exemplified with teretoxin Tv1 (Figure 2), which displays a conopeptide cysteine framework III but its disulfide connectivity is yet unseen in framework III conopeptides, resulting in a different fold from conopeptides [26]. It should be noted that three different connectivities of framework III conopeptides have been identified, demonstrating that this framework can lead a variety of folds [1].

Teretoxins appear to have similar biological effects on the nervous system to conopeptides. Indeed, a teretoxin isolated from *Terebra guttata* produced paralysis in polychaete worms [27] and injection of the venom from *Terebra subulata* and *Hastula hectica* in *Caenorhabditis elegans* elicited uncoordinated motions before paralysis [18,22,28]. Teretoxins from *Terebra argus* and *Terebra consobrina* primarily affected nicotinic acetylcholine receptors, but had no effect on voltage-gated potassium or sodium channels, as reported by Kendel [28]. It should be noted that venom extracts were used in these assays, and therefore Kendel speculates some low abundance toxins could still be targeting voltage-gated ion channels but would not have been noted in his experimental set-up [28]. Interestingly, Tv1 shares 85% sequence identity with conopeptide AsXVIA, which was postulated to target potassium channels [25]. 

Gorson et al. reported the identification of transcripts of putative pore-forming toxins identified from *Cinguloterebra anilis* and *Terebra subulata* [21]. These were named “tereporins” and determined to be closely related to conoporins from *Conus geographus* and *Conus radiatus*, as well as bearing similarities to actinoporins and echotoxins (discussed below) [21]. 

In summary, teretoxins have many similar features to conopeptides, but there are differences in cysteine frameworks and size [21,27]. Similar to *Conus*, analogues of conopressins and pore-forming toxins have also been found in Terebridae [21]. With approximately 300 different species of terebrids, and multiple new superfamilies of teretoxins identified from just a few species, there are a large number of novel teretoxins awaiting characterization and these toxins could display novel cysteine frameworks, three-dimensional structures, or biological activities [19,21]. 

### 2.2. Turridae 

**Figure 3:**
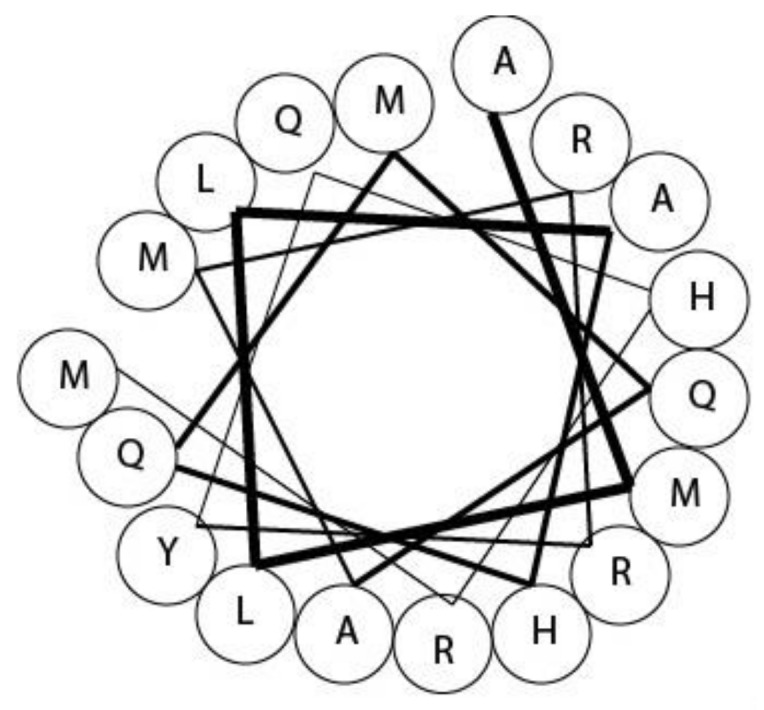
Helical wheel representation of *Polystira albida* turritoxin (PaIAa) residues 33–50, which form one of two predicted α-helixes.

Turridae is a large (estimated 4000 spp.) family of small, predominately deep-sea dwelling species, that belong to the superfamily Conoidea and are therefore related to terebrids and cone snails [16,21]. Originally this large group was classified as a single family; but, more recently the taxonomy of this family has become hazy with multiple subfamilies proposed and rejected [29,30]. In addition, turrids make up most of the diversity in the Conoidea superfamily, making this family a fascinating starting point for the discovery of new bioactive compounds [20,31,32]. Turrids have a venom duct, but because of their small size, it is highly difficult to extract toxins, limiting the research carried out into these species. Despite these limitations, it is estimated that there are more than 10,000 different turritoxins [32]. From analysis of the DNA extracted from turrid *Lophiotoma olangoensis*, it was found this species has the capacity to produce peptides similar to the conopeptides of the I and P gene superfamilies [32]. However, the genes that encode “turritoxins” in *Lophiotoma olangoensis* have very little sequence similarity with those of *Conus* [32]. Only one gene superfamily in *Lophiotoma olangoensis,* which encodes a subset of turritoxins, has clear similarity to conopeptides, more specifically to I-superfamily conopeptides [32]. The size of the peptides is also different, with turritoxin peptides containing 80–110 amino acids, whereas most conopeptides are typically 10–30 amino acids in length [32]. A similar study of *Gemmula speciosa* discovered several conopeptide-like precursors [20]. Some turritoxins have been shown by Edman degradation to be post-translationally modified like conopeptides, with some proline and glutamate residues being modified into hydroxyprolines and γ-carboxyglutamic acid, respectively [20]. The amino acid sequence of toxins displaying the same cysteine frameworks were described as highly divergent, suggesting turritoxins potentially represent a large pool of diverse bioactive compounds [20]. Nevertheless, the analysis of the venom of other turrid species produced contrasting results, with the venoms of *Polystira albida* and *Gemmula periscelida* lacking cysteine rich peptides, as determined by MALDI and MS [31]. It was found that the prevalent peptides isolated from each species were homologous and were composed of α-helical regions [31]. The afore-mentioned study only isolated one major component from each species of turrid [31]. These peptides were found to contain a high number of tyrosine, arginine, and especially methionine residues, which are probably responsible for the formation of the α-helices (Figure 3) [31]. It is likely these peptides form coiled-coil motifs, which could function as stabilizing agents in the absence of disulfide bonds [31]. These peptides, named PaIAa and GpIAa constitute a novel class, and are not similar to conopeptides or to other turritoxins; this type of peptide is structurally characterized by the methionine regions, “methionine zipper” [31]. 

The turritoxin ubi3a, isolated from *Lophiotoma bisaya,* produces tremors in mice on intracranial injection [33]. This turritoxin displays a cysteine pattern similar to the conopeptide cysteine framework III [33]. Despite this similarity, ubi3a has a disulfide connectivity that has not yet been described in conopeptides [33]. It has excitatory activity in mouse dorsal root ganglion neurons; the specific target receptor was not identified, but activity on the nicotinic acetylcholine receptors was ruled out [33]. 

### 2.3. Buccinidae—True Whelks

**Figure 4:**
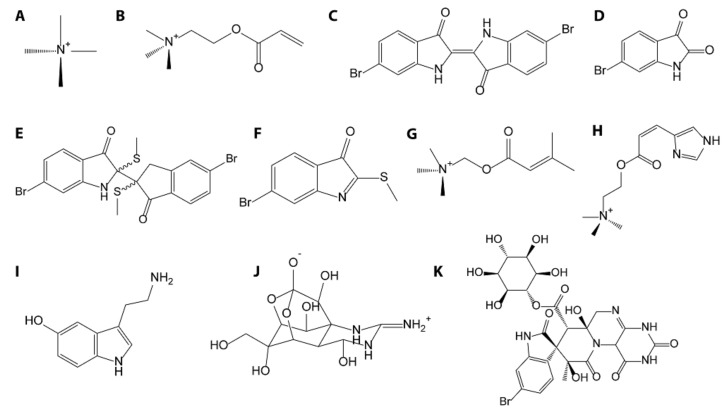
Small molecule compounds discussed in this review. (**A**) Tetramine, (**B**) acrylylcholine, (**C**) Tyrian purple, (**D**) 6-bromoisatin, (**E**) tyriverdin, (**F**) tyrindoleninone, (**G**) senecioylcholine, (**H**) urocanycholine (murexine), (**I**) serotonin (**J**) tetrodotoxin, and (**K**) surugatoxin.

One of the first reports of a neurologically active compound found in marine gastropod saliva was from a member of the Buccinidae family, *Neptunea antiqua*, when it was determined that the effects of the salivary gland extract were identical to the effects of neurine [34]. In multiple reports, it was found that diluted and buffered (pH 7) saliva was just as effective as the native acidic extract at paralyzing various species of gastropods, polychaetes, and urchins [34,35]. It was later reported that the saliva of some members of Buccinidae and Ranellidae families contains tetramethylammonium (or tetramine, Figure 4A), chemically similar to neurine, in concentrations high enough to cause food poisoning [36]. The marine gastropod species displaying high content of tetramine are *Buccinum leucostoma*, *Neptunea antiqua*, *Neptunea arthritica*, *Neptunea intersculpta*, *Neptunea kuroshio* and *Neptunea lyrata*, from the Buccinidae family, as well as *Fusitriton oregonensis* from the Ranellidae family (previously *Argobuccinum oregonense*) [37,38]. Other species in these families did not produce tetramine, therefore tetramine does not appear to be ubiquitous within Ranellidae or Buccinidae [39,40]. 

Tetramine (Figure 4A) acts by blocking nicotinic acetylcholine receptors, but human food poisoning cases caused by ingesting these gastropods are fortunately not serious because this toxin is easily degraded or cleared [14,37]. Indeed, symptoms typically vanish within 24 h at most, with no long-term complications reported [41,42]. An extensive study by Anthoni et al. [39] reports that only tetramine is responsible for toxic secretions of *Neptunea antiqua*. However, unpublished observations by West and Bowman point to additional bioactive substances in the salivary secretion of this species, though the specifics of these findings were not reported [3]. A closely related species, *Neptunea arthritica*, produces histamine and choline derivatives, in addition to tetramine [40,43].

A brief description of the bioactive extracts of *Cantharus tranquebaricus* demonstrated toxicity to brine shrimp, hemolytic activity, and some antimicrobial activity against *Vibrio cholerae* and *Proteus mirabilis* [44]. These extracts also appeared to have some proteolytic and fibrinolytic activity [44]. While the work was promising, additional studies using alternative methods are needed to support this report.

*Buccinum leucostoma* and *Buccinum undatum* were found to have acrylylcholine (Figure 4B), a neuromuscular blocking agent, in the hypobranchial gland [45]. *Buccinum* spp. are active predators of multiple species and although the reason for which these species use the compound is as yet undefined, it could be important for prey capture [46]. The salivary gland extract of *Buccinum schantaricum* was determined to be lethal to mice by intravenous injection [37], as mice injected with *Buccinum schantaricum* venom (0.01 mL/g body weight) exhibited convulsions and died within 5 min [37]. However, the extract lost its toxicity after heating to 80 °C for 5 min. Lethal activity was calculated in titer units and found to be only 4–8 [37], with titer being defined as the reciprocal of the highest dilution found to be lethal; for example, if a 1/10 (10%) dilution is lethal the corresponding titer unit would be 10. Large titer (in the thousands, for example) corresponds to greater toxicity. Low concentrations of tetramine were found in *Buccinum schantaricum*, considered by Shiomi et al. to be insignificant [37], suggesting that other compounds in the venom are responsible for the bioactivity. 

### 2.4. Cassidae—Helmet Shells

*Cassis tuberosa* mainly preys on sea urchins, with little regard for the urchin’s protective spines or toxins. Interestingly, when threatened, *Cassis tuberosa* will “spit” a clear liquid, evidently using its saliva as defense against other predators [47]. When the saliva was used in an in vivo study of urchins, it was found the saliva decreased the response of the urchin to sensory stimuli of all kinds, including light and touch [47]. Recovery was evidently complete, even after loss of all response to stimuli, except at the highest concentrations of saliva [47]. The unidentified compound was effective to some degree even at 400 ppm, with similar toxicity reported in two other species of *Cassis*: *Cassis madagascariensis,* and *Cassis flammea* [47]. 

### 2.5. Colubrariidae—Vampire Snail

The venom of *Cumia reticulata* (previously reported as *Colubraria reticulata*) was recently characterized using a RNA sequencing approach [48]. *Cumia reticulata* has a different feeding mechanism to other predatory gastropods mentioned previously; it feeds on the blood of fish that are resting nearby, leading to its common name, Vampire Snail. The venom is proposed to contain anesthetic peptide compounds, similar to the anemone potassium channel blocker ShK toxin. In addition, transcripts coding for putative inhibitors of primary hemostasis (ectonucleotide pyrophosphatase family), astacin metalloproteases, vasopressive proteins (angiotensive converting enzyme), and cytolytic porins were found [48]. The putative cytolytic porins display similarities to echotoxins, which are discussed in detail below [48]. Fascinatingly from an evolutionary perspective, a transcript coding for a peptide with a high similarity to some turripeptides was also discovered [48]. This study highlights the high diversity of proteinaceous components of predatory gastropod venoms. 

### 2.6. Muricidae—Rock Snails

Muricids have been used in traditional medicine over centuries for treatment of various medical disorders [49]. This family is most famous for the production of Tyrian purple, brominated indoles and their derivatives (see Figure 4D–F), which are traditionally used as a dye and produced in the hypobranchial gland of the gastropod. The indole compounds and their variants are relatively well-documented and investigated as potential anti-tumor and antibiotic agents, as reviewed by Bekendorff et al. [50,51,52,53]. Toxic choline esters have also been isolated from this family, found in the hypobranchial gland as a salt of brominated indole precursors [54]. Two substances, urocanycholine (murexine) (Figure 4H) and senecioylcholine (Figure 4G), have been isolated from the family Muricidae and have neuromuscular blocking properties through inhibition of nicotinic acetylcholine receptors [35,55]. Urocanycholine was isolated from *Hexaplex trunculus, Ocenebra erinaceus,* and *Bolinus brandaris* [45]. This is significant as choline compounds are neuromuscular inhibitors, found in other families, and are chemically related to tetramine [34,35,45]. In fact, over 50 species within the superfamily Muricoidea contain murexine or senecioylcholine (see Figure 4G,H) or both within the hypobranchial gland [55].

The salivary secretion of *Nucella lapillus* contains high levels of serotonin (an indole compound, see Figure 4I) and unidentified inhibitors of neural voltage-gated calcium channels [3,14]. The extracts from *Nucella lapillus* demonstrated effects characteristic of urocanycholine (see Figure 4H), and the active compound was later determined to be senecioylcholine [55,56]. However, there are also indications of another unidentified compound affecting nicotinic acetylcholine receptors [56]. It was found that this species secretes a glycoprotein that has several disulfide bonds, similar to many known toxins such as conopeptides, and might also be responsible for some of the bioactivity of the gland secretion [56,57]. *Nucella lapillus* has accessory salivary glands, in addition to the main salivary glands and it was proposed by West et al. [3] that these accessory salivary glands function as venom glands, being anatomically similar to cone snail venom glands. This proposal was not generally adopted, and these accessory salivary glands have remained “accessory” despite their important role in feeding. They are occasionally referred to as anterior salivary glands [3,14,58]. 

The extracts from *Acanthinucella spirata* salivary glands, which mainly preys on mussels, was found to induce flaccid paralysis in mussel tissues [59]. Carboxylic esters, as well as acetylcholine and butylcholine, were discovered in the salivary glands [59]. It was speculated that the carboxylic ester, acting on the same receptors as acetylcholine, is the paralytic component of the salivary glands [59]. In addition, proteolytic components were reported in *Chicoreus virgineus* also from the Murex family, but these were not further investigated [46]. 

The salivary gland extract from *Stramonita haemastoma* (formerly known as *Thais haemastoma*) is toxic to mice with LD_50_ of 43 mg/kg [60]. In various toxicity studies, the salivary gland extract appeared to act as a depressant on the central nervous system, causing vasodilation, hypotension, and bradycardia in anaesthetized cats [60]. However, an electrocardiogram recording revealed that this salivary extract was not damaging to the heart [60]. In isolated vertebrate ileum and duodenum, application of the salivary extract caused rapid contractions, which were not blocked by atropine, and it was concluded that this extract contains a toxin that acts by direct stimulation of smooth muscle fibers [60]. A previous report had demonstrated high concentrations of choline esters and senecioylcholine in the tissues of *Stramonita haemastoma* [55]. This may be responsible for the effects observed from the salivary gland extract of this species. However, if there is another toxic component in the salivary glands *Stramonita haemastoma*, it has not been characterized. 

### 2.7. Family Ranellidae

The family Ranellidae has been investigated only minimally. Here we will summarize what is known about Ranellidae, starting with the better-known species and then focusing on the genus Monoplex.

#### 2.7.1. Genus Charonia

*Charonia lampas* extracts cause instant paralysis of its main prey, the starfish *Patiriella regularis* by injection [45]. *Charonia lampas* toxins were unaltered by raising the temperature to 80 °C and were lethal to mice at 1 unit of titer [36]. Mice injected with the extract showed increased activity, spontaneous convulsion and jumping, and died of respiratory arrest within two minutes with blood found in the mouth and nose [36]. The saliva of *Charonia lampas* does not seem to induce paralysis of its main prey, the sea stars, during feeding, suggesting that the saliva does not contain analgesic compound [61]. Morton [61] suggested that the saliva of *Charonia lampas* is not venomous, despite contradictory reports by Shiomi et al. [36] describing lethal effects of this species’ salivary extract. Morton noted that the natural prey of *Charonia lampas* is the sea star *Astropecten polyacanthus,* which produces tetrodotoxin [61]. *Charonia lampas* has been reported to bioaccumulate tetrodotoxin, but tetrodotoxin cannot completely account for the activity observed in mice after injection [36,62]. Indeed, tetrodotoxin is a well-studied inhibitor of sodium ion channels, and associated symptoms in vertebrates include paralysis, abnormal heart rhythms, and respiratory arrest [62]. However, this toxin may not directly account for the increased activity or the blood found in the nose and mouth of the test animals.

The saliva of *Charonia tritonis* was first reported to paralyze its prey by Endean [45]. Recently, the composition of its saliva was analyzed from its salivary gland extract using a combined transcriptomics and proteomics approach [63]. It was found to contain cysteine-rich secretory proteins (CRiSPs), which were believed to have venom-like properties [63]. It was further speculated that these CRiSPs could be ion channel modulators, similar to their name-sake function in snake venom [63]. Metalloproteinases were also found in this study, and these proteins were also found in the venoms of cone snails, spiders, scorpions and platypus, which also use peptide toxins for defense [64,65]. In snake venom, metalloproteinases are responsible for breaking up tissue membranes and the extracellular matrix, which leads to hemorrhage [66,67]. In *Charonia tritonis*, the authors suggest these metalloproteinases may have a defensive function and call for further research into this area [63]. In a further study focusing on neuropeptides encoded by *Charonia tritonis* cerebral ganglion, authors found a transcript encoding a peptide similar to conopressins, which are expressed by cone snails *Conus geographus* and *Conus striatus* [68,69]. Conopressins have similar sequences to vasopressin and likely have neuroregulatory functions affecting multiple behaviors [68,70]. 

#### 2.7.2. Genus Monoplex

**Figure 5:**
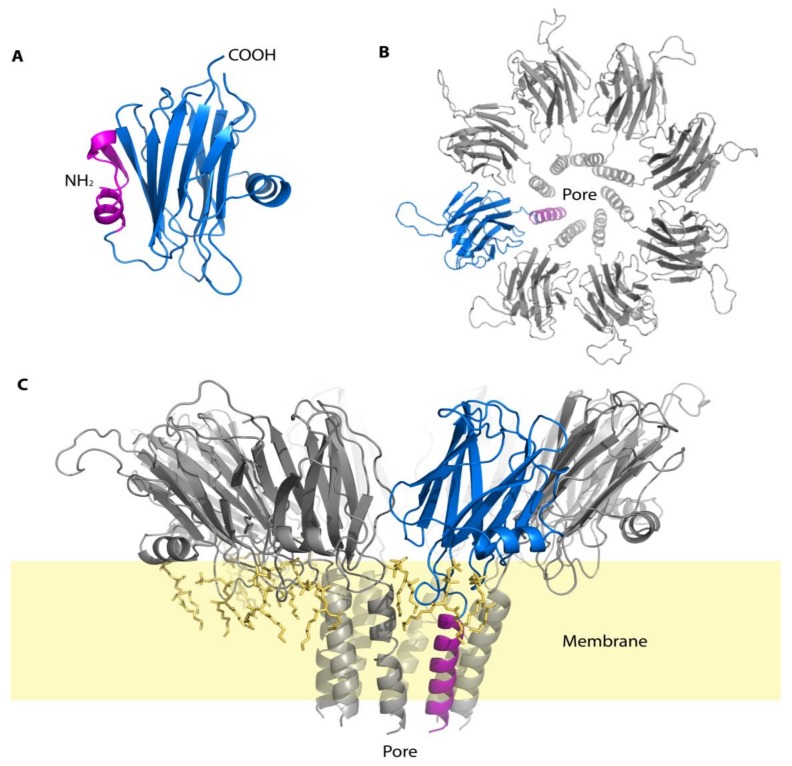
Putative three-dimensional structure of echotoxin 2 in monomeric (solution) and octameric (membrane-bound) forms. The structure was obtained by homology modeling using the crystallographic structure of fragaceatoxin C and generated using PyMol [73]. (**A**) Molecular model of monomeric, inactive echotoxin 2 (**B**) View from the extracellular side of the molecular model of echotoxin 2 in an octameric form (**C**) Side view of the molecular model of the octameric of echotoxin 2 embedded in a lipid bilayer (yellow). One of the monomer in each panel was represented in blue and its N-terminal active region shown in pink. This region change conformation depending if the monomer is in solution (**A**) or embedded in the membrane (**B**,**C**). The shaded region in (**C**) represent the approximate position of the membrane.

Monoplex species are active predators of other invertebrates, mostly other gastropods, sponges, or bivalves. To subdue their prey, bioactive secretions are produced from the paired salivary glands (Figure 5). These glands are segmented into anterior and posterior, which have different functions. It is believed that the different sections of the salivary glands secrete different proteins and acid [13,37,58,71]. It was also proposed that a chelating agent could be secreted together with the acid to assist in breaking down the shell of prey species [38,58]. *Monoplex intermedius* produces a toxin in its salivary gland that stimulates vertebrate and invertebrate nicotinic acetylcholine receptors in vitro [72]. The action of this toxin could be blocked by (+)-tubocurarine but not by atropine [72], suggesting that this toxin acts on nicotinic receptors but not on muscarinic cholinergic receptors. The same work determined similar activity from the salivary glands of *Monoplex gemmatus*, *Gutturnium muricinum* and *Monoplex nicobaricus* [72]. Although its physiological effects imitate acetylcholine, the nature of this toxin was not determined [72].

**Figure 6:**
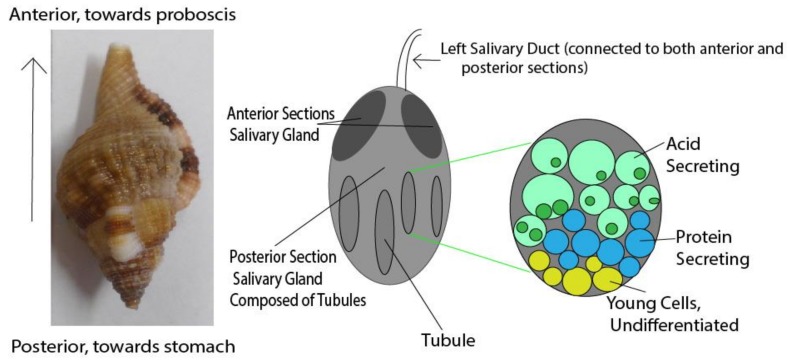
Diagram of the left salivary gland of *Cymatium*, showing the spatial differentiation within the blind tubules of the posterior salivary gland as described by Andrews et al. [58].

A fine structure analysis of cellular morphology in *Monoplex intermedius* revealed that the salivary glands are arranged into tubules (Figure 6) [58]. At the end of each tubule the cells do not appear to express distinctive traits, but more distal to the end of the tubule the cells secrete a light, heterogeneous product, likely made up of diverse proteinaceous components [58]. Continuing distally down the tubule, cell morphology changes again; cells becoming larger with more large vacuoles [58]. These vacuoles contain an acidic solution and the plasma membrane was found to have deep folds [58]. Researchers hypothesized that the undifferentiated cells at the ends of the tubules are the youngest, which later move down the tubule and specialize into protein secretion, finally becoming acid-secreting (Figure 5) [58]. 

Echotoxins are 25 kDa proteins isolated from the salivary gland of *Monoplex parthenopeus echo*, rich in glycine and alanine, and strongly inhibited by gangliosides [37]. Echotoxins were also found in the saliva of *Charonia tritonis* mentioned earlier in this review, which is the main predator of crown-of-thorns sea stars and is in the same family as *Monoplex parthenopeus echo* [63]. The main cause of death in mice injected with echotoxin was hemolysis, in contrast to the majority of other species discussed in this review with neurotoxic bioactivity [37]. The extract of *Monoplex parthenopeus echo* salivary glands were highly toxic with an LD_50_ of 82 μg/kg when injected intravenously into mice [37]. Assuming the effects on humans are comparable to those on mice, the equivalent is an estimated 6.4 mg = LD_50_ for 75 kg human. Echotoxins have similar biological activity to actinoporins, which are pore-forming hemolytic proteins isolated from Actiniidae and Stichodactylidae sea anemones [71,74]. Echotoxins also have similar size, basicity and N-terminal α-helix and aromatic “patch” to actinoporins [71]. The three-dimensional structure of echotoxins has not yet been experimentally determined, but with the knowledge of its primary structure and homology with actinoporins, its structure may be similar to known actinoporins (Figure 5) [71].

**Figure 7:**
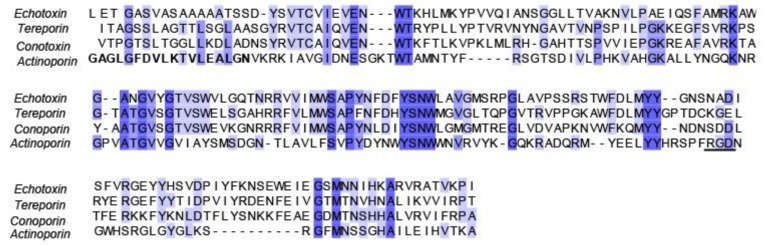
Sequence homology of echotoxin isolated from *Monoplex echo* with an actinoporin from *Actinia fragacea;* as well a conoporin from *Conus geographus* and a partial sequence of a tereporin from *Cinguloterebra anilis.* The RGD motif, present in the actinoporins but not the echotoxins, conoporins, or tereporins, is underlined. The N-terminal active region of the actinoporin is shown in bold. All sequences were retrieved from UniProt (accession numbers in Table A1) and aligned using Muscle [64].

Actinoporins and echotoxins have similar sequences, suggesting that they have similar activity, despite echotoxins lacking the arginine-glycine-aspartic acid (RGD) motif displayed by actinoporins (Figure 7) [71]. The RGD motif binds to integrin, which is a membrane protein, and this interaction possibly helps actinoporin to increase its local concentration at proximity of the targeted membrane. It is worth noting that conoporins, which are 36% identical to echotoxin 2 in sequence, have been identified in the venom of the cone snail *Conus consors* [75]. In addition, several other isoforms of conoporins with similar basicity to echotoxins have been reported in the venom of *Conus consors* and *Conus geographus* [76,77]. As mentioned previously, *Cumia reticulata* was reported to produce 62 different porins with similarities to echotoxin, which raises some interesting questions from the point of comparative biochemistry. It was surmised that the pore-forming toxins produced by this gastropod were necessary for permeating the blood vessels when feeding [48]. 

It has been proposed that the N-terminal α-helical region is essential for the biological activity of echotoxin 2 just as it is for actinoporins [71]. When actinoporins self-associate, eventually forming octamers, the N-terminus of each actinoporin monomer undergoes a conformational change and inserts into the cell membrane to form a pore (Figure 5) [73,74]. The similarities between echotoxin and actinoporins are shown in that they both interact with lipid components of cellular membranes, but probably use different mechanisms to recognize those membranes. Whereas actinoporins bind to sphingomyelin, echotoxins bind preferentially to gangliosides [71]. Gangliosides, unlike sphingomyelin, have no choline phosphate group, a negative charge, and are arranged asymmetrically with their carbohydrate moiety displayed on the extracellular side of the membrane [65].

### 2.8. Bioaccumulated Toxins—Selected Examples

There is a wealth of literature on the bioaccumulation of toxins (especially tetrodotoxin, organotins, and heavy metals) by marine gastropods, but this subject will not be extensively covered in this review, which primarily focuses on compounds produced by predatory gastropods. For a sample of the vast available literature on bioaccumualtion of various toxins relevant to the species mentioned in this review the reader is referred to Chau et al. [62], Costa et al. [78], Borysko and Ross [79], Choi et al. [80], Coelho et al. [81], Hwang et al. [82], and Zou and Kong [83]. For example, species of the Nassarius family, also known as the dogwhelks and closely related to Buccinidae, bioaccumulate toxins [83,84]. These species are often used as indicators of pollution, owing to their scavenging lifestyle and efficiency in bioaccumulating toxins [76,77,80,81,82,85]. Recently, the taxonomy of Nassaridae was rewritten using genetic barcoding and nuclear genetic markers, resulting in a call for a reassessment of the traditional classification based on morphology [86]. The formidable and often confusing taxonomy of these species, as well as lack of public information on which species are bioaccumulating or potentially producing toxins, makes these species generally dangerous for human consumption [76,77,80,81,82,83,85]. This makes the continuing popularity of these various species for sale as food, especially in China and Taiwan, rather puzzling [82,83]. The sale of toxic Nassarids is illegal in China, however, further research is needed to identify exactly which species bioaccumulate or produce toxins and whether these toxins are seasonal or not [83]. This information would be essential to ensure food safety of the general population*. Charonia lampas* has also been reported to bioaccumulate tetrodotoxin (see Figure 4J), and is discussed in further detail in the section on family Ranellidae [36,62].

The Japanese Ivory snail (*Babylonia japonica*) contains surugatoxin (see Figure 4K), an anti-nicotinic and ganglionic blocking neurotoxin [34,35]. Although it was initially believed that surugatoxin was produced by the snail, later work proposed that microbial activity (*Corynebacterium spp.*) was responsible for producing the precursors of surugatoxin in the gut of the snail [87]. 

## 3. Pharmaceutical and Biotechnological Applications

### 3.1. Venom-Derived Peptides

The applications of venom-derived disulfide peptides are numerous due to their specificity to various ion channels and receptors [88]. The intense biological selection pressure that produces a venom cocktail has been described as numerous successive clinical trials, weeding out ineffective compounds until only the most potent remain [88]. The approval of Prialt in the European Union and United States, as mentioned above, set the precedent for peptides as pharmaceuticals [10]. However, treatment of pain is far from the only application of bioactive peptides isolated from marine animals. ShK, a highly effective inhibitor of K_V_1.3 channels isolated from the sea anemone *Stichodactyla helianthus*, has been investigated for the treatment of several autoimmune disorders such as multiple sclerosis [88]. Conantokin-G, isolated from *Conus geographus*, is an NMDA antagonist that was found to have neuroprotective effects by reducing NMDA excitotoxicity [89,90]. In rat models of stroke, this ion-channel inhibitor significantly improved neurological recovery after ischemic injury [90]. The applications of highly selective and potent peptides are extremely broad, and an attempt to list all the potential biomedical applications alone is far beyond the scope of this review. For a review of conotoxins and their activities, the reader is referred to Akondi et al. and Halai and Craik [1,91]. The examples above are only a small fraction of the available research on venom-derived peptides. Multiple other peptides with high affinities for other channels have been described from Turridae and Terebridae and more await discovery, promising to be as fruitful as conopeptides [20,21]. 

### 3.2. Brominated Indoles—Muricidae 

Brominated indoles and their derivatives, as mentioned above, are produced by family Muricidae in the hypobranchial gland. These compounds are often found on the egg masses laid by members of this family and have been tested for antimicrobial and anticancer activity [50,51,53,92]. The compounds tyrindoleninone, tyriverdin, and 6-bromoisatin (see Figure 4) inhibited the growth of pathogens, both terrestrial and marine [92]. Tyrindoleninone and 6-bromoisatin, as well as other bromoisatin derivatives, were shown to inhibit multiple cancer cell lines, with minimal cytotoxicity to normal cells [51,52]. These compounds induce apoptosis in cancer cell lines and related indigo compounds have been patented for use in treatment of cancer [93,94,95]. In pre-clinical trials on mouse models of colorectal cancer, 6-bromoisatin induced apoptosis and prevented proliferation in cancer cells [93,94,95]. Other applications of these compounds and their relatives include use as anti-inflammatory agents [50,96,97,98]. In mouse model of acute lung inflammation, the hypobranchial gland extract from *Dicathais orbita* preserved lung tissue significantly better than the controls [98]. In fact, isatins and indole derivatives have been patented for treatment of inflammation and allergies [50,99]. Extensive research exists on the applications of brominated indoles, therefore the reader is referred to Benkendorff et al. for a further review of active components isolated from Muricidae [50]. 

### 3.3. Murexine

The use of murexine (see Figure 4G) as a muscle relaxant was investigated in the clinic by Erspamer and Glasser [100] as it appears to act on nicotinic acetylcholine receptors to produce paralysis. The action of murexine was more powerful than suxamethonium, the most similar compound tested in this experiment. The effects of murexine were temporary, only lasting 3 to 6 min [100]. Erspamer and Glasser initially suggested that murexine be investigated for anesthetic use and investigated murexine and related compounds for application as muscle relaxants [100,101]. However, some of the side effects of murexine were not desirable and its use in the clinic was discontinued [100,101]. 

### 3.4. Echotoxins 

The potential applications of pore-forming proteins such as echotoxins includes uses in the medical and analytical fields [102]. The delivery of proteins, peptides, and oligonucleotides to cells is severely limited by their inability to permeate the cell membrane [103] and pore-forming toxins may provide a platform for drug delivery on a cellular level. This could open a new field for pharmaceutical nano-particle based treatments [102,104]. The ability of pore-forming toxins to deliver cargo directly to the cytoplasm has already been demonstrated using disarmed anthrax toxin and streptolysin O [103,104,105,106,107]. A technique called suicide gene therapy could eventually employ pore forming toxins to deliver apoptotic agents directly into cancer cells [102]. 

Toxins can be used as therapies to kill malignant populations of cells when coupled to a targeting agent such as an antibody. The use of a toxin/antibody therapy has been labeled “conjugated drug delivery” where the antibody delivers the toxin directly to the target cell population, usually a malignant cancer cell [108]. Many of these conjugates using various bacterial-derived toxins and immunotoxins are already in clinical trials [108]. Echotoxins are effective at very low concentrations and are stable with changing pH [37,71], which may be desirable qualities when designing conjugated drug leads. 

Pore-forming toxins are also useful in analytical applications, namely nano-pore sensor research [109]. In brief, this technique can be used to determine properties of molecules by interactions with the pore (which has a known size and conductance), as well as sequestering molecules based on their permeability through the pore [109]. 

Whereas actinoporins in general bind to sphingomyelin, echotoxins in particular preferentially bind to gangliosides. Gangliosides are key factors in tumor angiogenesis, with expression of different gangliosides promoting or inhibiting angiogenesis [110,111]. Gangliosides are released by tumor cells into the surrounding matrix to stimulate vascularization [110,111]. An increased spread of tumors, stimulated by gangliosides, has been demonstrated for multiple cancer types such as lymphoma, melanoma, and neuroblastoma [110,111]. Echotoxin, while possibly having some potential for therapeutic use, has not yet been evaluated for prevention of angiogenesis.

There is also potential for use as a biomarker, as seen by the precedent of actinoporins and the cholera B subunit [111,112,113,114]. Cholera toxin B subunit, which similarly to echotoxins binds to gangliosides, has been used with a dye conjugate as a marker to identify ganglioside “rafts” [111,114]. In Alzheimer’s disease, ganglioside rafts play a role in the formation and accumulation of neurotoxin Aβ protein fibrils [111,114]. Despite many different potential applications of echotoxins, none of them have been investigated as of yet. 

## 4. Future Suggestions on Understudied Families

Very little is known about many families of predatory gastropods and there remain many which are not covered by this review. For example, there is no confirmed report of toxins in Mitridae. Unlike other species discussed in this review, Mitridae do not possess a venom gland or accessory salivary glands [115]. However, several observations suggest that these species are capable of producing valuable bioactive substances [114,115]. Some species within Buccinidae and Muricidae, discussed in other sections, are known to produce bioactive compounds in their hypobranchial gland. The Mitre hypobranchial gland has been noted to secrete a thick substance that oxidizes when exposed to air and turns purple; this secretion has an unknown function [115]. The Mitridae differ from other species in that they possess an epiproboscis in addition to the muscular proboscis found in other predatory gastropods and the salivary glands directly connect to this epiproboscis [116]. West described Mitridae specimen as using the epiproboscis to deliver salivary gland secretions to recalcitrant prey during feeding [116]. The diet of these species exclusively comprise of sipunculan marine worms [117]. Whereas several authors have speculated about the possibility of bioactive compounds from Mitridae, this possibility has never been explored [115,116]. 

Family Coralliophilinae (superfamily Muricoidea) are well-known predators of coral and apparently unaffected by the neurotoxins produced by corals [15]. This family may also be a source of useful bioactive compounds. In addition, other carnivorous or parasitic gastropod families could also be fruitful for bioprospectors. Less-well-known carnivorous superfamilies within Littorinimorpha and Neogastropoda include Vanikoroidea (parasitic), Turbinelloidea, and Volutoidea [17]. 

## 5. Conclusions

The potential for the discovery of active compounds in the venom of marine gastropods with applications in medicine, cellular biology or biotechnology is high and prompt further investigations. Apart from Conidae species, promising leads isolated from marine gastropod species have largely been ignored. New advances in technology, e.g., proteomics and transcriptomics, can now be used by researchers to rapidly catalog the protein and peptides expressed in the venom and saliva of marine gastropods, which is fundamental to help discover the compounds responsible for biological activity. Some compounds from marine gastropod besides the Conidae have been characterized and have shown potential biological, biotechnological, and/or medical applications. This suggests that most marine gastropods are a relatively untapped resource for new therapeutic leads.

## Figures and Tables

**Table 1:** Summary of species discussed in this article and their reported bioactive components or physiological effects.

Family	Species	Toxin
Conidae	*Various*	Conopeptides, CRiSPs, metallopreases
Terebridae	*Hastula hectica*	Teretoxins
	*Terebra subulata*	Teretoxins
	*Terebra argus*	Teretoxins
	*Terebra guttata*	Teretoxins
	*Cinguloterebra anilis*	Teretoxins, cytolytic proteins/actinoporins
	*Terebra consobrina*	Teretoxins, cytolytic proteins/actinoporins
Turridae	*Lophiotoma olangoensis*	Turritoxins
	*Polystira albida*	Turritoxins
	*Gemmula periscelida*	Turritoxins
Buccinidae	*Buccinum leucostoma*	Tetramine, cholines/murexines
	*Buccinum schantaricum*	Tetramine, unknown nervous system depressant? Stimulant?
	*Buccinum undatum*	Cholines/murexines
	*Neptunea antiqua*	Tetramine, unknown nervous system depressant
	*Neptunea arthritica*	Tetramine
	*Neptunea intersculpta*	Tetramine
	*Neptunea. kuroshiro*	Tetramine
	*Neptunea lyrata*	Tetramine
	*Cantharus tranquebaricus*	Antimicrobial, cytolytic activity?
Babylonidae	*Babylonia japonica*	Surugatoxin
Muricidae	*Hexaplex trunculus*	Choline esters/murexines, brominated indoles
	*Ocenebra erinaceus*	Choline esters/murexines, brominated indoles
	*Bolinus brandaris*	Choline esters/murexines, brominated indoles
	*Nucella lapillus*	CRiSPs? Choline esters/murexines, brominated indoles, unknown nervous system depressant? Stimulant?
	*Stramonita haemastoma*	Unknown nervous system depressant? Stimulant?
	*Acanthinucella spirata*	Cholines/murexines, unknown nervous system depressant
Cassidae	*Cassis tuberosa*	Unknown nervous system depressant
	*Cassis madagascarensis*	Unknown nervous system depressant
	*Cassis flammea*	Unknown nervous system depressant
Colubraridae	*Cumia reticulata*	Metalloproteases, cytolytic proteins/actinoporins
Ranellidae	*Charonia lampas*	Cytolytic proteins/actinoporins, unknown neurostimulant
	*Charonia tritonis*	CRiSPs, metalloproteases, echotoxins
	*Fusitriton oregonensis*	Tetramine
	*Monoplex intermedius*	Unknown neurostimulant
	*Monoplex parthenopeus echo*	Echotoxins

## Appendix A

**Table A1:** Sequences used to generate Figure 5, presented in the same order as shown in the figure. Accession numbers provided where applicable.

Organism	Toxin	Accession Number
*Monoplex echo*	Echotoxin 2	Q76CA2
*Cinguloterebra anilis*	Tereporin-Ca1	P0DN66
*Conus geographus*	Conoporin 5	W4VS02
*Actinia fragacea*	Delta-actitoxin-Afr1a	B9W5G6
